# Tumor Necrosis Factor and Splenectomy

**DOI:** 10.4274/tjh.2016.0040

**Published:** 2016-05-16

**Authors:** İrfan Yavaşoğlu

**Affiliations:** 1 Adnan Menderes University Faculty of Medicine, Division of Hematology, Aydın, Turkey

**Keywords:** Thalassemia, Tumor necrosis factor, splenectomy

## TO THE EDITOR

The article entitled “Effect of Tumor Necrosis Factor-Alpha (TNF-α) on Erythropoietin- and Erythropoietin Receptor-Induced Erythroid Progenitor Cell Proliferation in β-Thalassemia/Hemoglobin E Patients”, written by Tanyong et al. [1] and published in a recent issue of your journal, was quite interesting. Here we would like to emphasize some relevant points.

Splenectomy can increase the release of TNF-α and cell apoptosis in experimental and clinical studies in different diseases [2,3,4].

Increased serum TNF-α was reported in E/b-Thal patients, particularly after splenectomy [3,4]. In sickle cell disease presenting with functional asplenia, increased amounts of TNF-α, indicative of monocyte activation, and increased serum C-reactive protein levels were reported [5].

Banyatsuppasin et al. suggested the role of the spleen in controlling mononuclear phagocytic activity in E/b-Thal patients [6]. TNF-α play roles as an inducer and effector of monocyte activation [6]. Additionally, TNF-α returned to normal after 12, 6, and 3 months of deferiprone treatment [7]. Therefore, chelation treatment can affect apoptosis independently of splenectomy. It might be important to know the effect of chelation treatment and splenectomy on tumor necrosis factor in the study of Tanyong et al. [1] based on all these investigations stated above [2,3,4,5,6,7].
